# The Influence on Fracture Resistance of Different Composite Resins and Prefabricated Posts to Restore Endodontically Treated Teeth

**DOI:** 10.3390/polym15010236

**Published:** 2023-01-03

**Authors:** Saulo Pamato, Weber Adad Ricci, Milton Carlos Kuga, Eliane Cristina Gulin de Oliveira, João Carlos Silos Moraes, Marcus Vinicius Reis Só, Tamara Carolina Trevisan, Newton Fahl Júnior, Jefferson Ricardo Pereira

**Affiliations:** 1 Post-Graduate Program of Health Sciences, University of Southern Santa Catarina (UNISUL), Tubarão 89636-000, Brazil; 2 Department of Restorative Dentistry (FOAR), School of Dentistry at Araraquara, São Paulo State University (UNESP), Araraquara 01049-010, Brazil; 3 Department of Physics and Chemistry, School of Natural Sciences and Engineering, São Paulo State University (UNESP), 56 Brasil Avenue, Ilha Solteira 15385-000, Brazil; 4 Department of Endodontics, School of Dentistry, University Federal of Rio Grande do Sul (UFRGS), Porto Alegre 90000-000, Brazil; 5 Private Practice, Curitiba 59950-000, Brazil

**Keywords:** endodontically treated teeth, fiber post, fracture resistance, post-endodontic restoration, resin material

## Abstract

Recent formulations of resin-based composites have incorporated different combinations of materials. However, the mechanical and bonding behavior of these materials with intraradicular posts are unclear. This study aimed to evaluate the effect of light-cure and dual-cure resin composite posts on the fracture resistance of endodontically-treated teeth. Materials and Methods: Ninety extracted human upper canines were selected and randomly divided into nine groups (n=10): (G1) endodontically treated teeth without endodontic posts; (G2) glass-fiber post cemented with glass-ionomer cement; (G3) endodontic post by dual-cure composite resin (Rebilda DC); (G4) endodontic post by dual-cure composite resin (Cosmecore); (G5) endodontic post by dual-cure composite resin (Bis-Core); (G6) endodontic post by light-cure composite resin; (G7) glass-fiber post customized with flowable composite resin; (G8) glass-fiber post cemented with light-cure composite resin; (G9) glass-fiber post cemented with self-adhesive resin cement. After the post insertion, all specimens were subjected to mechanical (250,000 cycles) and thermocycling (6000 cycles, 5 °C/55 °C) and immediate loading at 45 degrees in a universal testing machine until fracture. The data were analyzed by one-way ANOVA and multiple comparisons using the Fisher LSD Method (*p* < 0 05). Results: The mean failure loads (±SD) for the groups ranged from 100.7 ± 22.6 N to 221.9 ± 48.9 N. The G1 group (without endodontic posts) had a higher fracture strength than all experimental groups (*p* < 0.001). Conclusions: Within the limitations, the light- and dual-cure post technique did not present lower fracture resistance values as compared to the conventional glass-fiber post.

## 1. Introduction

Endodontically treated teeth have often been considered more susceptible to fracture [1]. Although the periapical condition, arch position and morphological alterations due to endodontic treatment may influence the prognosis, the hypothesis of loss of structural integrity has justified an increase in the incidence of coronal and/or radicular fractures in endodontically treated teeth [2].

In an attempt to provide mechanical resistance to the coronal segment, allowing the functional and aesthetic rehabilitation of widely destroyed teeth [3], the use of intra-radicular posts has often been indicated. However, as of the advent of Pierre Fauchard in 1746, different systems have been suggested [4].

Although the use of intra-radicular posts does not provide support for or strengthening of the tooth, since this characteristic is a result of the quantity and quality of the remnant structure and surrounding alveolar bone, the different systems were evaluated to examine the incidence and mode of fracture [5]. Thus, adequate treatment planning is crucial for non-vital teeth [6].

**Figure 1:**
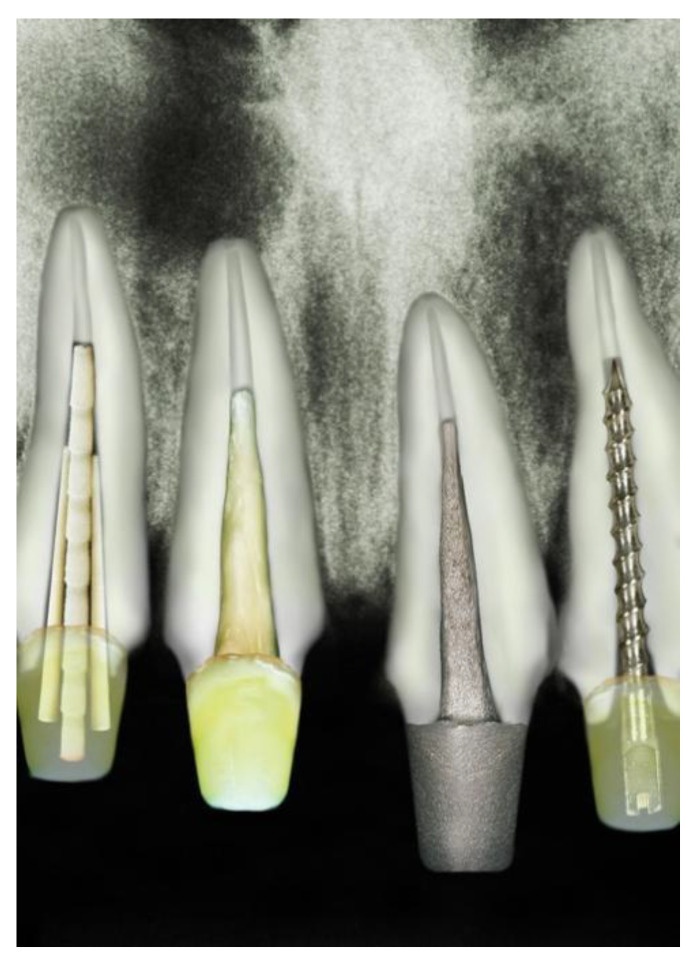
From left to right—Accessory Post, anatomical post, custom metal post, and stainless-steel post.

The conventional metallic cast posts have been gradually replaced by prefabricated posts, particularly those reinforced with glass fibers (Figure 1). Recently, the fabrication of intraradicular posts has changed from the exclusive use of rigid materials to materials with mechanical characteristics close to those of the dentin, reducing the risk of root fracture [7]. Examples of these materials are glass-fiber posts, which have the advantage of requiring low intraradicular thickness. They also have an elastic modulus close to dentin, and they may present better aesthetic properties due to their translucency [8].

Compared to metallic posts, prefabricated glass-fiber posts have better resistance to crack propagation [9], high translucency, and modulus of elasticity close to dentin, yielding excellent results [8]. However, this system has been the subject of debate because of the deterioration of the adhesion interface against functional loads, hygroscopic alteration susceptibility, and the need for a sufficient amount of dental structure [10].

Advancements in adhesive dentistry improved the fracture resistance of non-vital teeth. Using different curing modes, newer formulations of composite resins have incorporated different combinations of materials, helping to overcome the limitations of extended chairside time, depth of cure, reduced interlayer strength, and increased interfacial porosity [11]. However, the absence of a scientifically-based technique, capable of clarifying the mechanical behavior and the adhesive interaction of these hybrid compounds, associated or not with the presence of posts, encourages the conduction of further studies. Hence, the purpose of this study was to evaluate the influence of light-cure and dual-cure composite resin post on fracture resistance of endodontically treated teeth. The null hypothesis tested was that the use of intra-radicular posts made by light-cure and dual-cure composite resin did not influence the fracture resistance of endodontically treated upper canines.

## 2. Material and Methods

Ninety freshly extracted caries-free human upper canines with similar dimensions and anatomic structure were selected and stored in 0.9% physiologic saline solution with 1% thymol at room temperature. The teeth were examined under x4 magnification to remove remnants of periodontal tissue, and periapical radiographs were obtained to verify the absence of fractures and internal root resorption. Approval was obtained from the local ethical committee at the University of Southern Santa Catarina.

Coronal access were made with a diamond-coated spherical bur under continuous water-cooling and at high-speed rotation was used to form a triangular conservatory cavity. The working length was established in 0.5 mm short of the apex. The root canal was cleaned and shaped with a traditional technique to an ISO #50 K-file (Dentsply Maillefer, Ballaigues, Switzerland). Sodium hypochlorite (3%) was used during the instrumentation. The root canals were dried with absorbent paper points (Meta Biomed Co, Cheongju, South Korea) and obturated using the lateral condensation technique with gutta percha points (Meta Biomed Co, Cheongju, South Korea) associated with eugenol-free sealer (Dentsply Maillefer, Ballaigues, Switzerland). The cavity access was sealed with temporary restorative material (Coltene/Whaledent AG, Altstätten, Switzerland), and the teeth were stored in distilled water at room temperature for at least 72 h. The specimens were randomly divided into 9 groups (n = 10), according to the intra-radicular post (Table 1).

**Figure 2:**
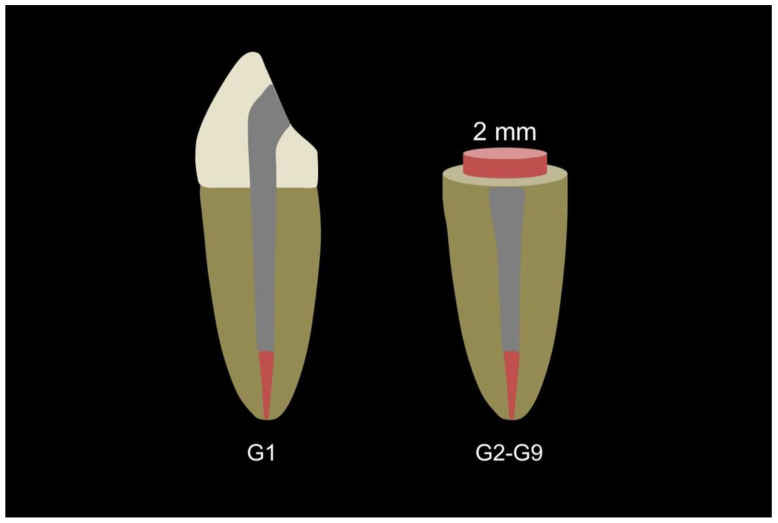
Control and experimental group preparation.

Other than the control group (G1), all specimens of the experimental groups were manually prepared by using rotary high-speed diamond instruments under copious water irrigation. A 2-millimetre-high complete ferrule was made to provide a protective effect (Figure 2). All specimens received posts and core crowns, except for those in the control group.

Post-spaces were prepared using a corresponding drill at low-speed rotation to achieve a post-space length of 10 mm (leaving at least 3 mm of gutta-percha in the apical third) in order to eliminate variables caused by post length differences. This preparation procedure permitted the fabrication of 10-mm posts for all specimens.

In group 2, the glass-fiber posts (D.T. Light-Post, Bisco, Schaumburg, IL, USA) were cemented using glass ionomer cement (Luting & Lining Cement, GC Corporation, Tokyo, Japan), according to the manufacturer’s instructions. In the G3–G6 experimental groups, the root canals were etched with 37% phosphoric acid (Ultra-etch, Ultradent, South Jordan, UT, USA) for 15 s, rinsed with an air/water spray, and gently dried with paper points. A thin layer of adhesive (Scotchbond Multi-purpose, 3M ESPE, St Paul, MN, USA) was applied with a microbrush, air-dispersed, and light-cured for 40 s (Valo, Ultradent, South Jordan, UT, USA) with the post placed inside the root canal to ensure post space. After post removal, the specimens received the injection of the dual resin material inside the root canal up to the coronary portion, as shown in Table 1. In the G7 and G8 groups, the prefabricated fiber posts were cleaned with 37% phosphoric acid (Ultra-etch, Ultradent, South Jordan, UT, USA), rinsed with an air/water spray, and gently humidified with silane for 60 s (Dentsply Sirona, Ballaigues, Switzerland). They were then customized by the root canal shape with flowable and conventional composite resin, respectively, and cemented with glass ionomer cement. Lastly, the specimens of G9 experimental group, after the root canal preparation, as previously described, self-adhesive resin cement was mixed and placed within the root canal using a periodontal probe, and the glass-fiber posts were cemented using adhesive systems. Each post was inserted into the root canal using finger pressure for 10 s, and the excess material was removed. The cores of G2, G6, G7, G8 and G9 groups were reconstructed using hybrid light-cure composite resin (Herculite, Kerr Corp., Orange, CA, USA) with conventional adhesive protocol.

In order to simulate moist conditions in the oral environment, all cementations occurred in a humid environment. The groups that received resinous material were light-cured at 1400 mW and a wavelength of 470 nm (Valo, Ultradent, South Jordan, UT, USA). Intra-radicular retainers were made, and each specimen was cemented with a metal crown using Luting & Lining Cement (GC Corporation, Tokyo, Japan).

The roots were embedded in self-polymerizing acrylic resin (JET, Classico, Sao Paulo, Brazil) leaving 2 mm of the coronal root surface exposed to imitate osseous support. After the roots were embedded in self-cure acrylic resin and the posts was built up or cementation (according to the group), all specimens were subjected to 250,000 mechanical cycling cycles with a load of 30 N on the palatal surface, (this simulated 8 years chewing aging) using a cylindrical puncher tip (2 mm diameter) from incisal/gingival direction, 3mm below the incisal edge, with a frequency of 2.6 Hz at an angle of 45 degrees from the long axis of the tooth (which simulated the occlusal contact of the antagonist tooth and chewing frequency in an angle class I relationship) [12], and then thermocycled in distilled water (6000 cycles; 5 °C/55 °C, 2-min dwell time) to simulated temperature modifications while eating. The mechanical and thermal cycling simulated 8 years of tooth aging. The specimens were stored at 37 °C in artificial saliva during the entire cycling stage.

All the specimens were then quasi-statically tested with a universal testing machine (Kratus K2000 MP, Dinamômetros KRATOS Ltd.a, Sao Paulo, Brazil) until fracture. The crosshead speed was 0.5 mm/min at an angle of 45 degrees from the long axis of the tooth. A compressive load was applied on a prepared notch (spherical with 1mm radius) on the palatal surface (done to apply all loads in the same point). Fracture patterns were examined under a light microscope and classified according to the type, location, and direction of failure. The normality data distribution was assessed using the Shapiro–Wilk test. Among the 9 groups, fracture load data were analyzed with 1-way ANOVA followed by multiple comparisons using the Fisher LSD Method (*p* < 0.05) using the SPSS statistic software (IBM, New York, NY, USA). The fracture load data were analyzed suing a statistical software (SPSS v18.0, IBM Corp, Armonk, NY, USA). A post hoc power analysis (=05) was done to determine the minimum sample size.

## 3. Results

The post hoc power analysis revealed that the power of the present study for each group was one. The highest fracture resistance scores were observed for the control group (G1), while the lowest ones were recorded for group G4. Fracture resistance data registered for each group can be observed in Table 2.

As described in the Table 3, one-way ANOVA analysis revealed significant differences between the groups (*p* < 0.001). The Fisher LSD Method revealed statistically significant differences between the control group (non-post) and all other experimental groups (*p* ≤ 0.037), as well as between G4 and G2, G3, G5, G8 and G9 (*p* ≤ 0.01), and G6 and G8 (*p* = 0.023). As shown in Table 2, significant differences were not identified between the other group combinations.

Fracture levels are shown in Table 4.

## 4. Discussion

This study investigated the effect of light-cure and dual-cure composite resin posts on fracture resistance of restored endodontically treated teeth. The null hypothesis that the use of intra-radicular posts made by light-cure and dual-cure composite resin did not affect the fracture resistance of endodontically treated maxillary canines was rejected. However, when those techniques were compared to the conventional protocol (prefabricated fiber post) only one flowable dual-cure composite resin presented lower fracture resistance values as compared to the others. These results may provide less root structure loss, increasing the fracture resistance of endodontically treated teeth [8].

The results of the fracture resistance shown in G1 (control group) presented the highest scores among the groups (221.9 ± 48.9). These findings are in accordance with previous studies that reported the harmful effects of extensive restoration procedures or the non-use of minimally invasive protocols [13,14]. Therefore, a conservative endodontic access design must be preferred in order to reduce the risk of fracture in non-vital teeth [15]. Whereas endodontic treatment reduces only 5% of the fracture tooth resistance, the marginal ridge region loss (as MOD cavities) reduces nearly 75% of the initial fracture tooth resistance, reducing the cuspal deflection [16]. Therefore, the amount of remnant tooth structure after endodontic access appears to be a crucial factor for the prognosis of endodontically treated teeth and in the decision-making to use intra-radicular posts [17].

Although the scores of endodontically treated teeth without post seem to discourage the placement of an intra-radicular retainer, the in vitro analysis may not reflect accurately intraoral conditions over time to make a comfortable clinical decision. On the other hand, if the prevalence of both caries and periodontal diseases has declined, the non-carious cervical lesions are now receiving attention [18]. Pathologies that affect the hard dental tissue such as abrasion or abfraction, while weakening tooth structure, directly affect the long-term survival of the tooth. This concept is supported by other studies that concluded that placement of an intra-radicular post becomes interesting when the amount of dentin decreases [17,19]. In addition, a randomized clinical trial has demonstrated that failure risk was significantly lower in non-vital restored teeth with fiber post when compared to the non-post group [20].

Since the high-stress concentration region is in the cement enamel junction, the failure mode is the most important parameter to compare restoring techniques [7]. Corroborating findings of previous studies, the majority of fracture patterns examined under a light microscope were favorable [21,22]. These findings may suggest that the similarities presented between elastic modulus of the dentin (18.6 GPa), fiber-glass post (20 GPa), and resin-based materials employed (12 GPa) are able to prevent the microcracks propagation and favor the dissipation of occlusal load along the root structure and support tissues, besides decreasing the stress generated in the residual tissue [23].

**Figure 3:**
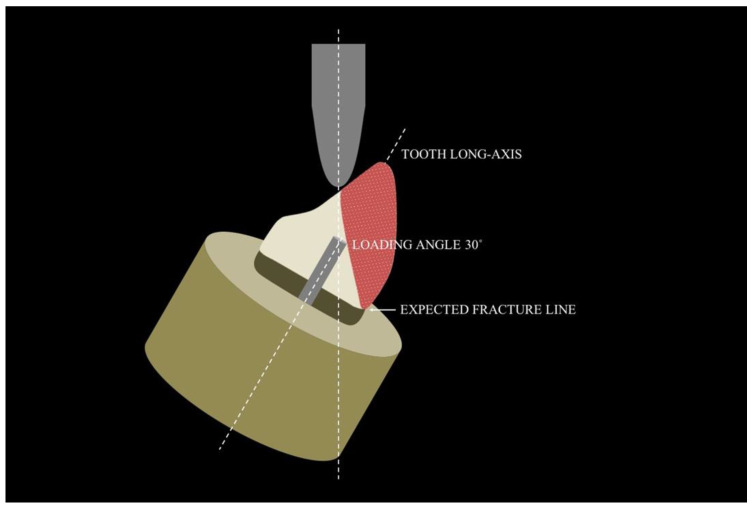
Expected fracture lines according to the loading angles with 30 degrees from the long-axis of the root.

Some clinical studies have confirmed the long-term clinical performance of the prefabricated fiber post [24,25,26]. In contrast, a previous study described that the loading angle may affect the failure mode of the specimens [27]. In cases where the loading angle was in 30 degrees to the long-axis of the root, the cusp did not receive support from the post and more catastrophic (unfavorable) fractures have been expected to occur (Figure 3). 

**Figure 4:**
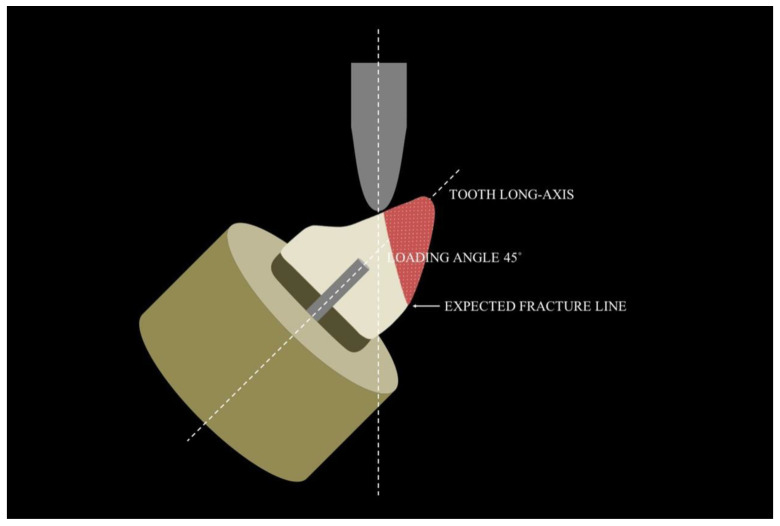
Expected fracture lines according to the loading angles with 45 degrees from the long-axis of the root.

**Figure 5:**
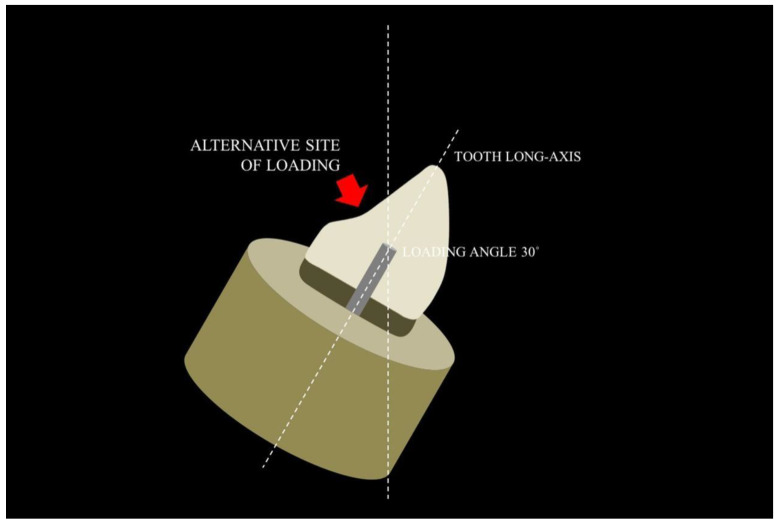
Expected fracture lines according to the loading angles with load applied on the alternative site.

On the other hand, when loading angles were changed to 45 degrees (Figure 4) in relation to radicular long-axis, the fracture line moves to above the cement enamel junction, resulting in an expressive number of composite resin fractures. According to these authors, if the load had been applied on the alternative site (Figure 5), the number of vertical root fractures would be greater, possibly due to a rotation of the post inside the root canal [2,20,28,29]. Although in vitro tests seem to provide conflicting results, the systematic review and a large number of others clinical studies indisputably generate the most reliable evidence [2,20,28,29].

Although no statistical difference was observed between the glass-fiber post group (G2) and the others groups with post (G3–G9), the use and effect of light-cure and dual-cure composite resin as a post can be questioned. From a mechanical perspective, the advantage of using a highly viscous material (G3 and G4) instead of the prefabricated fiber post is to avoid the technical procedure of post placement, which would exclude the need for root canal enlargement. Based upon this suggestion, the lowest scores presented by the Comescore™ DC dual-cure resin composite (G4) may be associated with their high elasticity modulus, resembling the mechanical behavior of cast or zirconia post and cores [30,31].

In general, the preservation of tooth structure is important for increasing the fracture resistance of endodontically treated teeth. The ideal intra-radicular post material should provide adequate stress distribution, being able to reduce tensile and compressive failures, and provide high reparability. In view of this, the dual-cured resin composite posts proved to be useful for this purpose. Nevertheless, extrapolating these in vitro results indiscriminately to a clinical situation may be a dangerous practice because it would be impossible to simulate all oral conditions. Further investigations, such as push-out bond strength and other clinical research, are needed to understand the complete effect of using light-cure and dual-cure composite resin posts in restorative dentistry.

Within the limitations of this in vitro study, the placement of conventional glass-fiber post did not improve the fracture resistance of endodontically treated teeth as compared to the light-cure or dual-cure post technique. Despite the fact that no post group presented high fracture resistance values, the great incidence of cervical lesions should be considered in clinical decision-making regarding the placement of intra-radicular posts, since tooth longevity depends on the amount of remnant tooth structure and the capability of the restorative materials to replace the lost hard tissue. The strength point of this study is that there are many possibilities to restore endodontically treated teeth but the professional must consider their choice based on the prognosis of the treatment. The most important objective for teeth in this situation is to preserve dental structure as much as possible, so as to provide less root structure loss and increase the fracture resistance of endodontically treated teeth.

## 5. Conclusions

The placement of conventional glass-fiber posts did not improve the fracture resistance of endodontically treated teeth compared to the light-cure or dual-cure post technique. Despite the fact that no post group presented high fracture resistance values, the great incidence of cervical lesions should be considered in clinical decision-making about intra-radicular post placement, since tooth longevity depends on the quantity of tooth remaining and the capability of the restorative dental materials to replace lost hard tissue.

## Figures and Tables

**Table 1:** Groups and materials used for restorative procedures.

Group	Material
G1	Control group (non-post)
G2	Prefabricated fiber post cemented with glass-ionomer cement
G3	Dual-cure composite resin post (Rebilda DC, Voco)
G4	Dual-cure composite resin post (Cosmecore, Cosmedent)
G5	Dual-cure composite resin post (Bis-Core, Bisco)
G6	Composite resin post (Herculite, Kerr)
G7	Prefabricated fiber post with flowable composite resin (Tetric N-flow, Ivoclar Vivadent) cemented with glass-ionomer cement
G8	Prefabricated fiber post cemented with composite resin (Herculite, Kerr)
G9	Prefabricated fiber post cement with self-adhesive resin cement (BisCem, Bisco)

**Table 2:** Fracture load (N) of different groups.

Group	Load (N) Mean ± SD
G1	221.9 ± 48.9 ^A^
G2	154.8 ± 26.6 ^B,D^
G3	151.3 ± 56.4 ^B,D^
G4	100.7 ± 22.6 ^C^
G5	170.2 ± 41 ^B,D^
G6	134.8 ± 33.6 ^B,C^
G7	169.6 ± 35.8 ^B,C,D^
G8	179.6 ± 45.2 ^D^
G9	170.2 ± 45.4 ^B,D^

Statistically different means (*p* < 0.05) are indicated by different superscript letters.

**Table 3:** One-way ANOVA analysis of failure loads.

Source of Variation	Sum of Squares	*df*	Mean Square	F	*p*
Between groups	71302,311	8	8912,789	5,323	<001
Within groups	113857,299	68	1674,372		
Total	185159,61	76			

**Table 4:** Distribution of failure modes.

Failure Mode	Experimental Group								
	G1	G2	G3	G4	G5	G6	G7	G8	G9
1. Complete decementation of post-and-core and crown	-	-	1	-	2	3	2	3	2
2. Fracture composite resin foundation	-	9	6	6	4	4	8	7	8
3. Coronal fracture	5	-	2	3	2	1	-	-	-
4. Root crack	4	-	-	-	-	-	-	-	-
5. Horizontal root fracture	1	1	1	1	2	2	-	-	-
Failure favorable/unfavorable total	9/1	9/1	9/1	9/1	8/2	8/2	10/0	10/0	10/0

## Data Availability

Not applicable.
